# Different BCR/Abl protein suppression patterns as a converging trait of chronic myeloid leukemia cell adaptation to energy restriction

**DOI:** 10.18632/oncotarget.13319

**Published:** 2016-11-12

**Authors:** Silvia Bono, Matteo Lulli, Vito Giuseppe D'Agostino, Federico Di Gesualdo, Rosa Loffredo, Maria Grazia Cipolleschi, Alessandro Provenzani, Elisabetta Rovida, Persio Dello Sbarba

**Affiliations:** ^1^ Department of Experimental and Clinical Biomedical Sciences “Mario Serio”, Università degli Studi di Firenze, Florence, Italy; ^2^ Centre For Integrative Biology (CIBIO), Università degli Studi di Trento, Trento, Italy

**Keywords:** transcriptional regulation, translational regulation, post-translational regulation, hypoxia, glucose shortage

## Abstract

BCR/Abl protein drives the onset and progression of Chronic Myeloid Leukemia (CML). We previously showed that BCR/Abl protein is suppressed in low oxygen, where viable cells retain stem cell potential. This study addressed the regulation of BCR/Abl protein expression under oxygen or glucose shortage, characteristic of the *in vivo* environment where cells resistant to tyrosine kinase inhibitors (TKi) persist. We investigated, at transcriptional, translational and post-translational level, the mechanisms involved in BCR/Abl suppression in K562 and KCL22 CML cells. *BCR/abl* mRNA steady-state analysis and ChIP-qPCR on *BCR* promoter revealed that *BCR/abl* transcriptional activity is reduced in K562 cells under oxygen shortage. The SUnSET assay showed an overall reduction of protein synthesis under oxygen/glucose shortage in both cell lines. However, only low oxygen decreased polysome-associated *BCR/abl* mRNA significantly in KCL22 cells, suggesting a decreased BCR/Abl translation. The proteasome inhibitor MG132 or the pan-caspase inhibitor z-VAD-fmk extended BCR/Abl expression under oxygen/glucose shortage in K562 cells. Glucose shortage induced autophagy-dependent BCR/Abl protein degradation in KCL22 cells. Overall, our results showed that energy restriction induces different cell-specific BCR/Abl protein suppression patterns, which represent a converging route to TKi-resistance of CML cells. Thus, the interference with BCR/Abl expression in environment-adapted CML cells may become a useful implement to current therapy.

## INTRODUCTION

Chronic myeloid leukemia (CML) is a hematopoietic stem cell-derived and progenitor-driven myeloproliferative disorder that may progress from a clinically manageable chronic phase to an incurable “blastic” phase [1]. CML is characterized by the t(9;22)(q34;q11) reciprocal translocation and the consequent generation of a chimeric *BCR/abl* oncogene, encoding for a 210-kDa fusion oncoprotein (BCR/Abl), endowed with constitutive tyrosine kinase activity, which is essential for CML onset, maintenance and progression [1]. The BCR/Abl oncoprotein activates several downstream pathways, responsible for the inhibition of programmed cell death, induction of cell proliferation, block of cell differentiation, and loss of cell adhesion [2]. Consequently, BCR/Abl represents the primary target of CML therapy [3], which is based on tyrosine kinase inhibitors (TKi) targeting BCR/Abl enzymatic activity. TKi, however, although extremely effective in inducing remission of disease, are unable in most cases to prevent relapse [4].

Low oxygen (O_2_) tension is a critical aspect of the metabolic *milieu* where stem cells (SC) are long-term maintained [5]. In “physiologically hypoxic” SC niches, low O_2_ tension offers a selective advantage to the maintenance of hematopoietic SC with respect to less immature progenitors [6, 7]. We also found that low O_2_ restrains the clonal expansion of SC without blocking their cycling, thereby contributing to maintain SC potential [8].

Cancer SC (CSC), like normal SC, most likely rely on metabolically-restricted environments for the regulation of the balance between self-renewal/maintenance and clonal expansion/differentiation [9, 10]. CSC homing within SC niches is indeed the best candidate mechanism to sustain the so-called minimal residual disease (MRD) and thereby the risk of relapse of the disease even in patients who brilliantly responded to antiblastic treatments [4]. Thus, conditions enabling CSC homing within SC niches are worth being characterized to try to optimize the long-term outcome of therapy.

As far as CML is concerned, we previously demonstrated that the leukemia stem cell (LSC) phenotype is preserved under metabolic restrictions (O_2_ and/or glucose shortage) which suppress BCR/Abl protein expression [11, 12]. Metabolically-selected LSC are thereby refractory to Imatinib mesylate (IM) and most probably to all other BCR/Abl-targeting TKi. This points to the metabolic regulation of CML cell phenotype, namely the presence or absence of expressed BCR/Abl protein, as an important factor controlling the onset of TKi-resistant MRD and the related relapse of disease [4].

The understanding of the regulation of BCR/Abl protein expression under metabolic pressure suffers from significant gaps. In this study, we addressed the effects of CML cell incubation under O_2_ or glucose shortage and determined how these metabolic constraints drive BCR/Abl protein suppression. We identified multiple cell-specific BCR/Abl suppression patterns, each cell line exhibiting a characteristic combination of transcriptional, translational and post-translational mechanisms.

## RESULTS

### Effect of oxygen and/or glucose shortage on CML cell survival and growth

**Figure 1:**
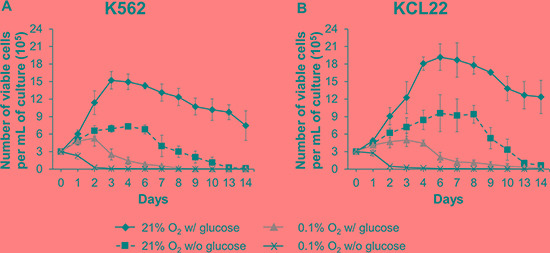
Effects of oxygen and/or glucose shortage on CML cell survival and growth K562 (**A**) or KCL22 (**B**) cells were plated at 3 × 10^5^ cells/mL and incubated at 21% O_2_ w/ glucose (♦) or 21% O_2_ w/o glucose (■), or at 0.1% O_2_ w/ glucose (▲) or 0.1% O_2_ w/o glucose (**X**). Viable cells were counted by trypan blue exclusion at the indicated times. The graphs show means ± SD of 7 independent experiments. K562 cells: *p* ≤ 0.01 (♦) compared with (■), (▲, days 2–14), (**X**); *p* ≤ 0.01 (■) compared with (▲, days 3–8). KCL22 cells: *p* ≤ 0.01 (♦) compared with (■), (▲, days 2–14), (**X**); *p* ≤ 0.01 (■) compared with (▲, days 2–9) (two-tailed Student's *t* test).

We previously demonstrated that incubation of K562 cells for 7 days in O_2_ shortage results in BCR/Abl protein suppression, which parallels glucose exhaustion in culture medium [12]. In the study reported here, we addressed the effects of O_2_ (0.1%) or glucose shortage separately, comparing K562 with KCL22 CML cells, aiming at the characterization of molecular mechanism driving BCR/Abl protein suppression. As shown in Figure 1A, under standard culture conditions (21% O_2_ w/ glucose), K562 cell number increased about 5-fold over the first 3 days of incubation, to decrease thereafter as an effect of culture crowding. Under glucose and, even more, O_2_ shortage, cell number increase was significantly reduced. The combined O_2_/glucose shortage was a too stringent condition, zeroing the number of viable cells on day 2 of culture. Thus, we decided to exclude this condition from further experiments. Figure 1B shows that KCL22 cells behaved likewise, although with a 2–3 day delay of cell number peaking and decrease when compared to K562 cells. The Annexin V/PI assay showed a small amount of cell death/apoptosis during the time frame used in our further experiments (Supplementary Figure S1).

### BCR/Abl protein is suppressed in CML cells under oxygen or glucose shortage

**Figure 2:**
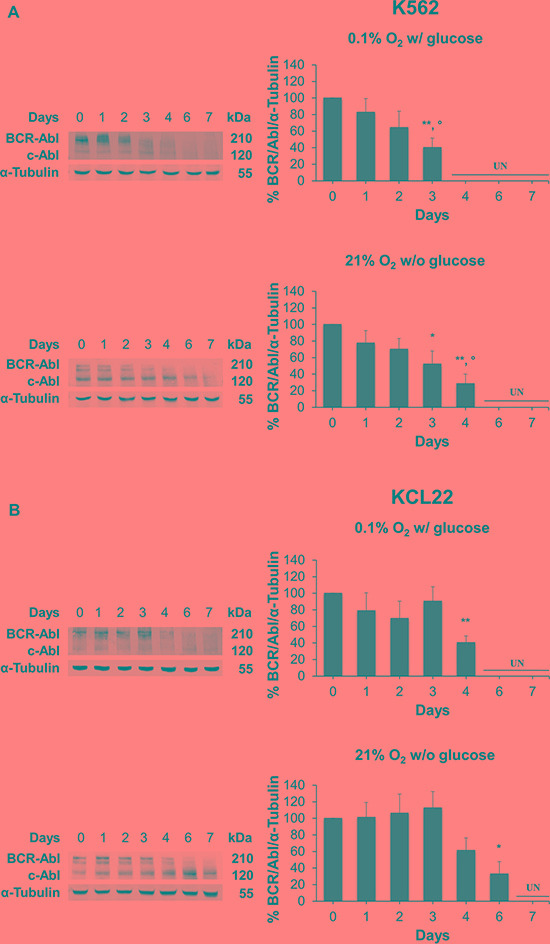
BCR/Abl protein suppression under oxygen or glucose shortage K562 (**A**) or KCL22 (**B**) cells were incubated at 0.1% O_2_ in standard medium (top panels) or 21% O_2_ in the absence of glucose (bottom panels) for the indicated times. The levels of BCR/Abl protein were determined by Western blotting using α-Tubulin as loading control. Band intensity was quantified using the Odyssey software. Data were normalized with respect to the corresponding α-Tubulin band intensity and expressed as percentage of time 0 (day 0) value. UN: undetectable. Histograms represent the mean + SD of 3 independent experiments; **p ≤ 0.01, *p ≤ 0.05 compared with time 0; °*p* ≤ 0.05 compared with day 1 (two-tailed Student's *t* test).

We then assessed by Western blotting a 7-days kinetics of BCR/Abl protein levels in K562 and KCL22 cells incubated in low O_2_ or in the absence of glucose. Either O_2_ (top panels) or glucose (bottom panels) shortage determined a time-dependent suppression of BCR/Abl protein in K562 cells. This suppression was faster under O_2_ restriction (Figure 2A) with respect to that under glucose restriction. KCL22 cells behaved likewise (Figure 2B), although suppression occurred more slowly than in K562 cells. These cell line-specific differences of BCR/Abl protein suppression kinetics, when evaluated together with those relative to the reduction of CML cell number (Figure 1), seem to indicate that this reduction followed BCR/Abl suppression, in keeping with previous conclusions [12].

### *BCR/abl* mRNA is differentially expressed under oxygen or glucose shortage

**Figure 3:**
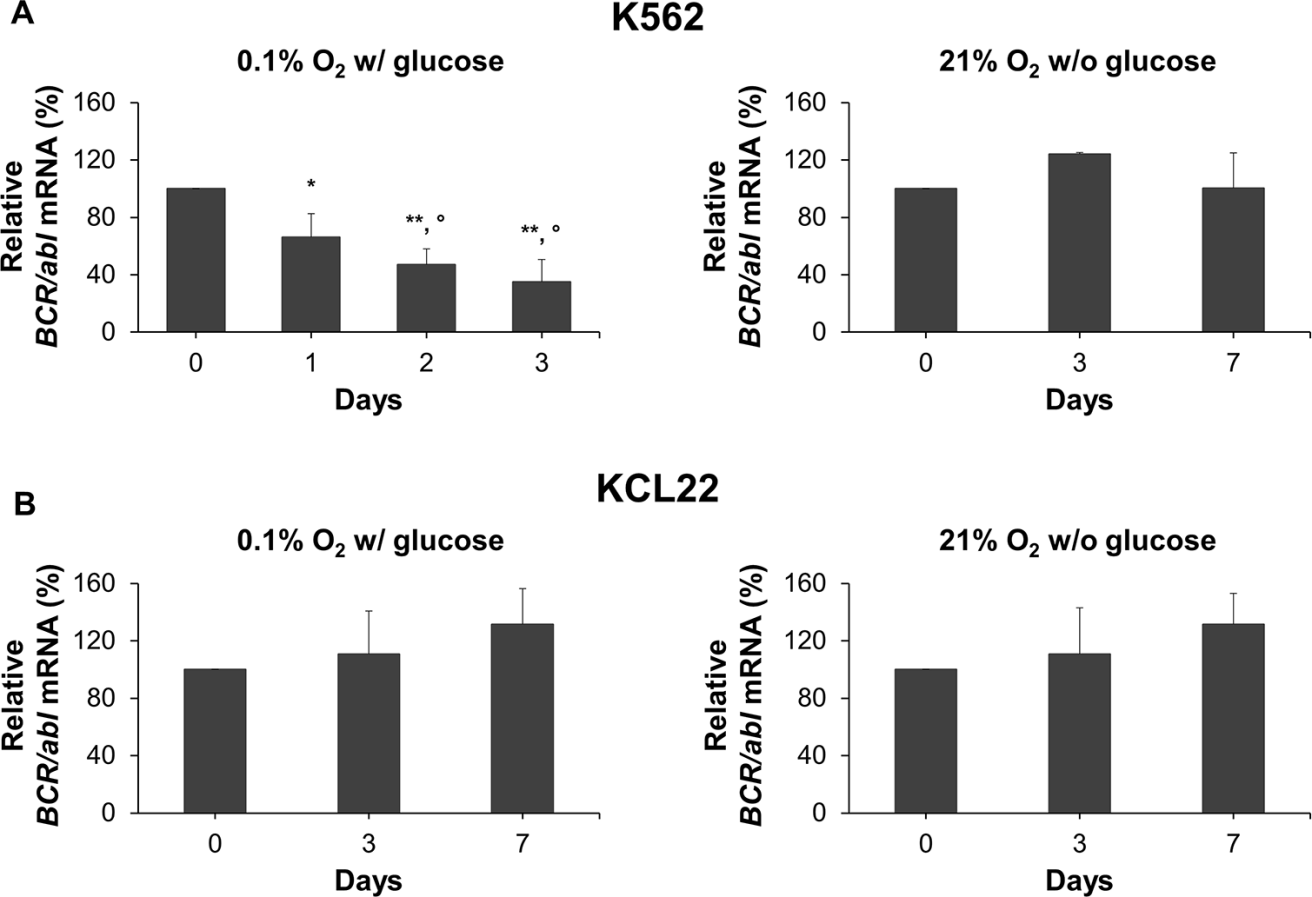
*BCR/abl* mRNA expression under oxygen or glucose shortage K562 (**A**) or KCL22 (**B**) cells were incubated at 0.1% O_2_ in standard medium (left panels) or at 21% O_2_ in the absence of glucose (right panels) for the indicated times. *BCR/abl* mRNA was measured by qPCR and its quantity expressed as percentage of time 0 value. Data were normalized within each experiment using different housekeeping genes and the results from different experiments mediated. These genes, chosen because their expression did not change under the experimental condition used, were: for O_2_ shortage, *GAPDH*, *18S*, β-actin and *GUSB* for K562 cells and *GAPDH*, *18S*, *EIF2a* and β-2 *microglobulin* for KCL22 cells; for glucose shortage, *GAPDH* and *β-actin* for either cell line. Data are mean + SD of 3 independent experiments; **p ≤ 0.01 compared with day 0; °*p* ≤ 0.05 day 2 and day 3 compared with day 1; °*p* ≤ 0.05 day 3 compared with day 2 (two-tailed Student's *t* test).

The mechanisms driving BCR/Abl protein suppression were deepened analyzing multiple levels of *BCR/abl* gene expression under metabolic restriction. We evaluated first the total *BCR/abl* mRNA relative amount. Figure 3A (left panel) shows a significant reduction of *BCR/abl* mRNA in K562 cells as early as day 1 of incubation in low O_2_, to decrease further in the following days. On the contrary, glucose shortage did not alter *BCR/abl* mRNA level in K562 cells (Figure 3A, right panel). As far as KCL22 cells are concerned (Figure 3B), neither O_2_ (left panel) nor glucose (right panel) shortage led to the reduction of *BCR/abl* mRNA level over 7 days of incubation. Overall, these results suggested the existence of cell line- and metabolic condition-specific mechanisms of BCR/Abl protein suppression under energy restriction.

### Transcriptional regulation of *BCR/abl* mRNA in K562 cells under oxygen shortage

**Figure 4:**
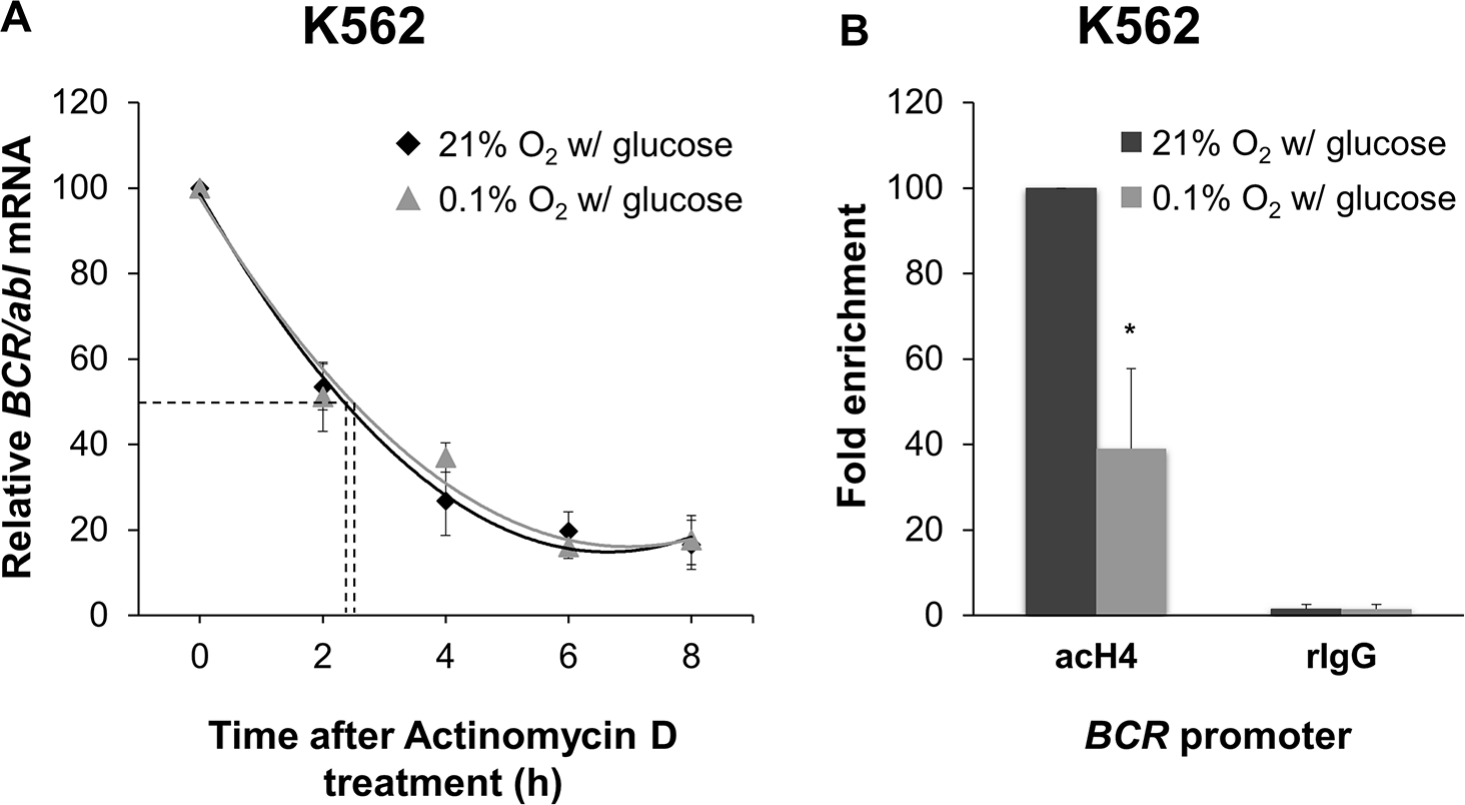
Effects of oxygen shortage on *BCR/abl* mRNA stability and *BCR* promoter activity (**A**) K562 cells were incubated at 0.1% or 21% O_2_ in standard medium for 8 hours in the presence of the transcriptional inhibitor actinomycin D (5 μg/mL). Cells were harvested every 2 hours and qPCR for *BCR/abl* was performed. *GAPDH* was used as housekeeping gene. *BCR/abl* quantity was normalized by the ratio of *BCR/abl* to *GAPDH* mRNA and expressed as percentage of time 0 (h 0) value. Data are mean ± SD of 3 independent experiments. Interpolating curves were determined by polynomial best fit (R^2^ = 0.982 for 0.1% O_2_ and 0.994 for 21% O_2_). (**B**) K562 cells were incubated at 0.1% or 21% O_2_ in standard medium for 36h and lysed. ChIP was performed using an antibody against acetylated histone H4 (acH4) and a control rabbit IgG (rIgG) followed by qPCR for the *BCR* promoter. Histograms represent the relative quantification of DNA recovered from IP. Data were normalized for input values and expressed as fold-enrichment with respect to control IgG; the fold-enrichment value obtained for 21% O_2_ was arbitrarily set to 100%. Data are mean + SD of 3 independent experiments; *p ≤ 0.05 (two-tailed Student's *t* test).

On the basis of the results of Figure 3A, we determined whether the decrease of *BCR/abl* mRNA in K562 cells incubated in low O_2_ could be attributed to an altered mRNA stability or to a reduced transcriptional activity. Cells were treated with the transcriptional inhibitor actinomycin D and *BCR/abl* mRNA levels were monitored by qPCR every 2 hours for 8 hours after treatment (Figure 4A). *BCR/abl* mRNA half-life, as determined by polynomial best fit of data obtained, was 2.5 hours for incubation in low O_2_ *versus* 2.4 hours in standard conditions, indicating that O_2_ shortage does not significantly affect *BCR/abl* mRNA half-life. Therefore, we determined, by evaluating *BCR* promoter activity, if transcriptional activity was reduced in K562 under O_2_ shortage (Figure 4B). *BCR* promoter controls the transcription of both *BCR* and *BCR*/*abl* genes [13]. K562 cells were incubated for 36 hours in low O_2_ or under standard conditions and then ChIP-qPCR was performed to detect acetylated histone H4 at the *BCR* promoter. H4 acetylation is generally associated with chromatin unfolding and transcription initiation [14, 15]. O_2_ shortage significantly reduced the level of H4 acetylated at the *BCR* promoter in keeping with a reduced expression of *BCR/abl* mRNA.

### Translational control of BCR/Abl protein under oxygen or glucose shortage

To evaluate if translational machinery also regulates BCR/Abl protein expression under energy restriction, we performed the SUnSET (SUrface SEnsing of Translation) assay and polysome profiling analysis.

**Figure 5:**
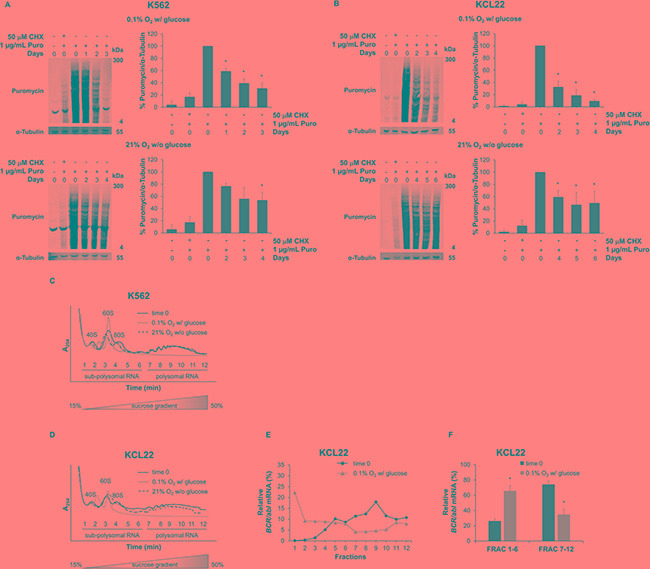
Effects of oxygen or glucose shortage on BCR/Abl translation K562 (**A**) or KCL22 (**B**) cells were incubated at 0.1% O_2_ in standard medium (top panels) or at 21% O_2_ in the absence of glucose (bottom panels) for the indicated times and treated with 1 μg/mL of puromycin (Puro) for 6 hours. Cell extracts were separated by denaturing electrophoresis and analyzed by Western blotting with a monoclonal antibody to puromycin (12D10), using α-Tubulin as loading control. The intensity of entire lanes was quantified using the Odyssey software, in relation to puromycin signal. Data were normalized with respect to the corresponding α-Tubulin band intensity and expressed as percentage of time 0 value for puromycin-treated cells. Histograms represent the mean + SD of 3 independent experiments; *p ≤ 0.05 compared with puromycin-pretreated time 0 (two-tailed Student's *t* test). K562 (**C**) or KCL22 (**D**) cells were incubated at 0.1% O_2_ in standard medium or at 21% O_2_ in the absence of glucose. Prior to cell lysis, ribosomes were immobilized on the mRNA by the treatment with the translation elongation inhibitor cycloheximide at 100 μg/mL final concentration for 15 min. Cytosolic extracts were fractionated over a 15 to 50% sucrose gradient, and the absorbance at 254 nm (A_254_) of sub-polysomal (1 to 6) and polysomal (7 to 12) fractions was continuously monitored. The positions of the 40S, 60S, 80S and polysomal peaks for time 0 are indicated. Results are representative of 3 independent experiments. qPCR analysis of relative *BCR/abl* mRNA levels in single cytoplasmic RNA fractions (**E**) and pooled sub-polysomal (FRAC 1-6) and polysomal (FRAC 7-12) fractions (**F**) of KCL22 cells subjected to 0.1% O_2_ in standard medium; data are mean + SD of 3 independent experiments; *p ≤ 0.05 compared with time 0 (two-tailed Student's *t* test).

The SUnSET assay allows to determine the mRNA translational rate based on incorporation of puromycin into nascent polypeptide chain [16]. To determine the optimal concentration of puromycin for our experiments, we assayed puromycin incorporation and we found that 1 μg/mL produced the best signal, with a greater proportion of labeled high molecular weight proteins, indicating minimal protein truncation and degradation (Supplementary Figure S2) [17]. Figure 5A and 5B show that puromycin incorporation decreased progressively upon either O_2_ (top panels) or glucose (bottom panels) shortage in both K562 and KCL22 cells, indicating that protein synthesis is restrained during energy restriction. To evaluate the specificity of the assay, we also pretreated cells with the translation inhibitor cycloheximide that, as expected, completely blocked puromycin incorporation.

Further, *BCR/abl* mRNA translation was deepened by investigating its polysomal loading. A change in the association of mRNA with polysomes is indicative of changes in its translation state [18]. We performed sucrose gradient fractionation of cytoplasmic sub-polysomal (representative of non-translating monosomes) and polysomal (representative of actively-translating poly-ribosomes) RNA from K562 or KCL22 cells incubated under O_2_ or glucose shortage (Figure 5C and 5D). The polysome profile in lysates of untreated cells (time 0) showed three defined peaks (40S, 60S, and 80S) in the less dense fractions (1 to 6) and increasing levels of polysomal RNA (*i.e.* numbers of associated ribosomes) in the denser fractions (7 to 12). Low O_2_ conditions resulted in a shift to free ribosomal subunits and a marked decrease of polysomal RNA in both K562 and KCL22 cells; polysomal profiles from glucose shortage conditions showed no qualitative differences with respect to time 0. RNA fraction analyses in KCL22 cells showed that low O_2_ reduced the polysomal loading of *BCR/abl* mRNA with respect to time 0, while increasing the sub-polysomal fractions (Figure 5E). These data were also supported by the quantification of the *BCR/abl* mRNA level in collected sub-polysomal (fraction 1 to 6) and polysomal (fraction 7 to 12) compartments (Figure 5F). The results indicated that the poly/sub-poly ratios decreased significantly (*p* ≤ 0.05) under O_2_ shortage in KCL22 cells. Glucose shortage, on the contrary, did not reduce these ratios significantly in KCL22 cells (data not shown). Finally, in K562 cells, the association of *BCR/abl* mRNA with the actively-translated fractions did not change in either O_2_ or glucose shortage (data not shown).

Overall, these data highlighted the strong contribution of translational regulation to the reduced production of BCR/Abl protein under low oxygen conditions in KCL22 cells. By contrast, in K562 cells, alterations of BCR/Abl translation did not seem to be involved, despite the evident reduction of protein synthesis under either O_2_ or glucose shortage.

### Post-translational control of BCR/Abl protein suppression under oxygen or glucose shortage

**Figure 6:**
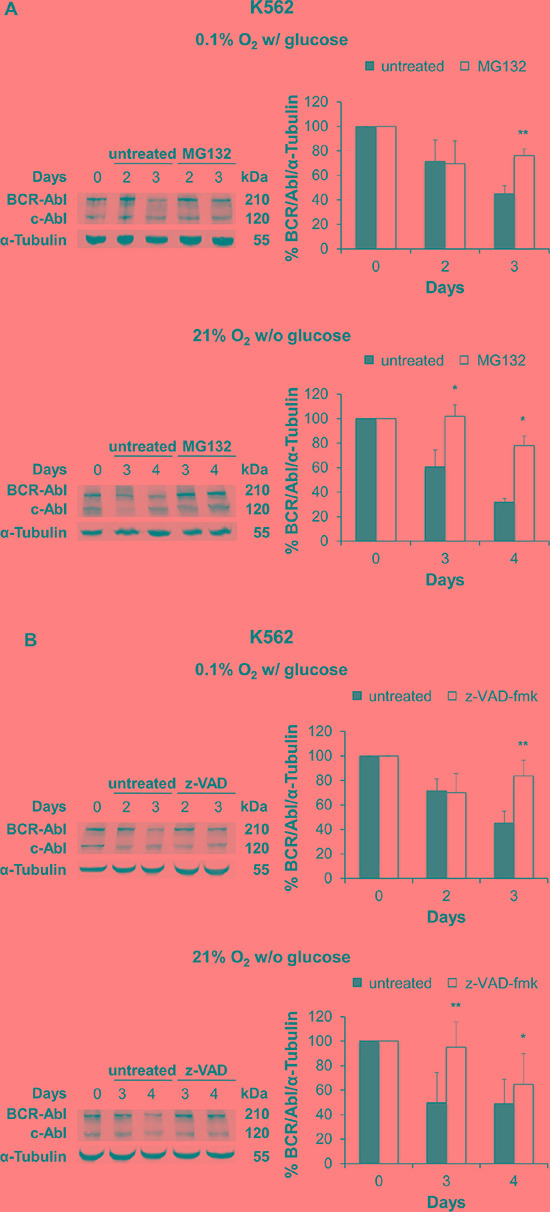
Effect of proteasome or caspase inhibition on BCR/Abl protein suppression under oxygen or glucose shortage K562 cells were incubated at 0.1% O_2_ in standard medium (top panels) or 21% O_2_ in the absence of glucose (bottom panels) for the indicated times, treated with the proteasome inhibitor MG132 (0.3 μM) (**A**) or the pan-caspase inhibitor z-VAD-fmk (50 μM) (**B**) for the indicated times and lysed. BCR/Abl protein expression was determined by Western blotting using α-Tubulin as loading control. Band intensity was quantified using the Odyssey software. Data were normalized with respect to the corresponding α-Tubulin band intensity and expressed as percentage of time 0 (day 0) value. Histograms represent the mean + SD of 3 independent experiments; **p ≤ 0.01, *p ≤ 0.05 compared with untreated (two-tailed Student's *t* test).

To explore the contribution of post-translational control mechanisms to BCR/Abl protein suppression, we also determined the involvement of proteasome machinery. K562 and KCL22 cells were incubated under oxygen or glucose shortage in the presence or the absence of the proteasome inhibitor MG132. As shown in Supplementary Figure S3, we preliminarily determined the optimal concentration of MG132 for each cell line: 0.3 μM for K562 cells and 0.1 μM for KCL22 cells. These MG132 concentrations inhibited the ubiquitin-proteasome machinery (Supplementary Figure S3A and S3D) and did not affect cell survival (Supplementary Figure S3B and S3E) or induce apoptosis (Supplementary Figure S3C and S3F). Under either O_2_ (Figure 6A, top panel) or glucose (Figure 6A, bottom panel) shortage, the maintenance of BCR/Abl protein in K562 cells was significantly enhanced in cultures treated with MG132. Thus, proteasome activity was strongly involved in BCR/Abl protein suppression under energy restrictions in K562 cells. On the other hand, in KCL22 cells, MG132 treatment had no effects (data not shown).

To determine whether BCR/Abl protein suppression was also due to the activation of caspases, we assessed the effect of the pan-caspase inhibitor z-VAD-fmk. Inhibition of PARP cleavage showed that 50 μM z-VAD-fmk protected K562 and KCL22 from apoptosis under either experimental condition (Supplementary Figure S4). Figure 6B shows that, under O_2_ (top panel) or glucose (bottom panel) shortage, the maintenance of BCR/Abl protein in K562 cells was significantly enhanced in cultures treated with z-VAD-fmk. Thus, caspases contributed to BCR/Abl protein suppression under energy restriction in K562 cells. Differently, in KCL22 cells, z-VAD-fmk treatment had no effects (data not shown).

The combined treatment with MG132 and z-VAD-fmk in K562 cells showed no synergistic or additive effects (Supplementary Figure S5).

**Figure 7:**
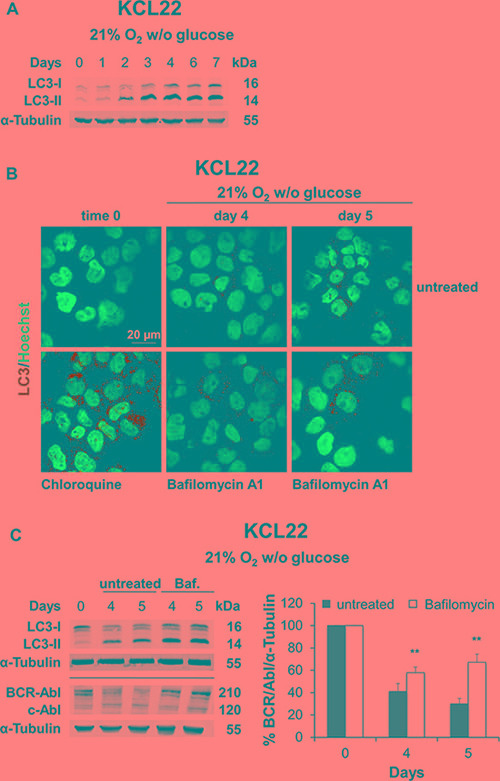
Role of autophagy in BCR/Abl protein suppression under glucose shortage KCL22 cells were incubated at 21% O_2_ in the absence of glucose for the indicated times. (**A**) LC3-I and LC3-II levels were determined by Western blotting, using α-Tubulin as loading control. (**B**) Following treatment with the late-stage autophagy inhibitor Bafilomycin A1 (2 nM) added on day 3, cells were incubated for further 1 or 2 days and the expression of LC3 was then assessed by immunofluorescence. Treatment with 100 μM Chloroquine for 24 h was used as a positive control of autophagic flux inhibition. Nuclei were stained by Hoechst 33342. (**C**) Following treatment with Bafilomycin A1 on day 3 (Baf. or open columns), cells were incubated for further 1 or 2 days and lysed. LC3-I and LC3-II (top blot), and BCR/Abl (lower blot) protein levels were determined by Western blotting using α-Tubulin as loading control. BCR/Abl band intensity was quantified using the Odyssey software (right panel). Data were normalized with respect to the corresponding α-Tubulin band intensity and expressed as percentage of time 0 (day 0) value. Histograms represent the mean + SD of 3 independent experiments; **p ≤ 0.01 compared with untreated (two-tailed Student's *t* test).

In order to assess whether autophagy was involved in BCR/Abl protein suppression under energy restrictions, we first analyzed the microtubule-associated protein light chain 3 (LC3), the main marker of autophagosomes, in K562 and KCL22 cells subjected to O_2_ or glucose shortage. Autophagy is characterized by the accumulation of the cleaved and lipidated form of LC3 (LC3-II) [19]. Glucose shortage induced marked accumulation of LC3-II in KCL22 cells, starting from day 2 (Figure 7A), while only a slight LC3-II increase occurred in K562 cells (Supplementary Figure S6A, right panel). On the contrary, O_2_ shortage showed no LC3 activation in either cell line (Supplementary Figure S6A left panel: K562 cells, and S6B: KCL22 cells), indicating that autophagy was not involved in BCR/Abl protein suppression in low O_2_. To confirm that LC3-II increase in KCL22 cells under glucose shortage was actually due to an active autophagic flux, we evaluated the LC3-II level in the presence or absence of bafilomycin A1. This is a specific inhibitor of vacuolar-type H^+^-ATPase, which inhibits autophagy at a late stage by increasing the lysosomal intracellular pH, thus preventing the fusion of autophagosomes and lysosomes and the consequent degradation of autophagic proteins [20, 21]. Thus, a further LC3-II increase in the presence of bafilomycin A1 reflects an actual induction of autophagy [19]. Confocal microscopy with LC3 immunofluorescence revealed an increased punctate staining in KCL22 cells maintained under glucose shortage for 4–5 days with respect to time 0 (Figure 7B, upper panels). In bafilomycin A1-treated cells this effect was enhanced, as expected; the treatment with high-dose chloroquine was used as a positive control for the inhibition of autophagic flux (Figure 7B, lower panels). LC3-II protein increase after bafilomycin A1 treatment was further confirmed by Western blotting (Figure 7C, upper blot). To directly link autophagy to BCR/abl protein suppression under glucose shortage, we determined the BCR/Abl protein level in the presence or absence of bafilomycin A1. As shown in Figure 7C (lower blot and histogram), BCR/Abl protein level was significantly increased in bafilomycin A1-treated KCL22 cells with respect to untreated control. In K562 cells, on the contrary, bafilomycin A1 treatment did not determine BCR/Abl protein maintenance (data not shown). These results demonstrated the autophagic degradation of BCR/Abl protein under glucose shortage in KCL22 cells.

## DISCUSSION

**Figure 8:**
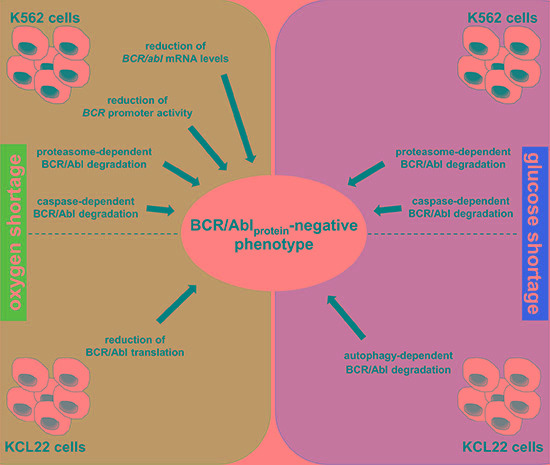
Summary of the multi-layer BCR/Abl protein suppression pattern under energy restriction

This work provides a mechanistic explanation to the previous findings of ours that BCR/Abl protein is suppressed under energy restriction [11, 12]. We found here that, in K562 cells, O_2_ shortage reduced *BCR/abl* mRNA levels as well as *BCR/abl* promoter activity, while either O_2_ or glucose shortage led to proteasome- and caspase-dependent BCR/Abl protein degradation. On the other hand, in KCL22 cells, O_2_ shortage reduced BCR/Abl translation, while glucose shortage induced autophagy-dependent BCR/Abl protein degradation (Figure 8). Thus, a complex scenario emerged where the two cell lines behaved quite differently, yet converging to BCR/Abl protein suppression. These differences likely derive from the fact that K562 and KCL22 cells, although originating both from CML blast-crisis patients, exhibit a different phenotype, as witnessed by the quite different proteome profiles [22]. Our evidences led to conclude that energy restriction induces cell line-specific, multi-layer BCR/Abl suppression patterns, each cell line exhibiting a characteristic combination of transcriptional, translational and post-translational mechanisms of suppression. This underscores the necessity of CML cells subjected to energy restriction to undertake a process leading to BCR/Abl protein suppression regardless of the molecular mechanisms involved.

A top-down conceptual process commanded to explore first the role of *BCR/abl* transcription. O_2_ shortage caused a significant reduction of *BCR/abl* mRNA levels as well as *BCR/abl* promoter activity in K562 cells. Reduced *BCR* promoter activity has been found upon myeloid differentiation from hematopoietic SC to common myeloid progenitors [23, 24]. This activity is maintained in chronic-phase CML and overactivated in blast crisis, leading to BCR and BCR/Abl overexpression [13]. Furthermore, *BCR/abl* transcription is controlled by the Sp1 (Specificity protein 1) and Myc transcription factors and via histone H4 hyperacetylation [25–28]. Noteworthy, Myc and Sp1 are involved in the transcriptional program leading to hypoxic adaptation [29, 30]. Our evidences indicated that, while glucose, differently from O_2_, shortage did not determine the reduction of *BCR* promoter activity in K562 cells, both conditions reduced cell number in culture. This suggests that reduction of *BCR* promoter activity is not directly linked to block of proliferation, but is rather a specific consequence of hypoxic signaling, possibly related to some of the above-mentioned transcriptional or epigenetic regulators.

KCL22 cells under O_2_ shortage underwent reduction of BCR/Abl protein translation. Translation is a very energy-demanding process [31], which is in general shut down under oxygen or glucose shortage [32]. Anyhow, non-canonical or cap-independent mechanisms of translation initiation ensure synthesis of proteins whose expression has to be preserved under stress conditions [33]. Consistent with these evidences, we found that both K562 and KCL22 cells underwent overall translational inhibition under O_2_ or glucose shortage. Surprisingly, BCR/Abl translation rate was reduced only in KCL22 cells, and only under O_2_ shortage. On this basis, we hypothesize that BCR/Abl translation could bypass canonical cap-dependent mRNA recruitment. However, this hypothesis requires further investigations.

At the post-translational level, we explored the involvement of proteasome, caspases and autophagy in BCR/Abl degradation. We demonstrated that in K562 cells, under either O_2_ or glucose shortage, BCR/Abl protein suppression relies on proteasomal degradation. Proteasomal degradation of immature BCR/Abl protein has been observed in the absence of its Hsp90-mediated proper folding, following protein recognition by the E3-ubiquitin ligase CHIP (Carboxyl terminus of Heat shock cognate protein 70 (Hsc70)-Interacting Protein) and the involvement of Bag1 (Bcl-2-associated athanogene-1) protein. In addition, the E3-ubiquitin ligase c-Cbl (Casitas b-lineage lymphoma) induces degradation of mature and phosphorylated BCR/Abl protein [34]. Moreover, it has been demonstrated that β-TrCP (β-Transducing repeat Containing Protein) upregulation results in enhanced BCR/Abl ubiquitination and its consequent degradation in K562 cells [35]. As for caspases, we showed their role in BCR/Abl protein suppression in K562 cells under either O_2_ or glucose shortage, in keeping with what previously described for K562 cells undergoing erythroid differentiation [36]. The ubiquitin-proteasome system (UPS) has an important role in apoptosis [37]. However, depending on the cell type, interference with UPS protects from or triggers apoptosis, via caspase degradation or mitochondrial cytochrome C release and caspase activation, respectively [38, 39]. Thus, a fine balance between UPS and caspases exists to regulate cell fate decisions [39]. Finally, we found that autophagy led to BCR/Abl protein degradation in KCL22 cells under glucose shortage. Autophagy is part of the cell's survival response to stress, including energy shortage [40]. Taken together, our results indicated that post-translational mechanisms are heavily involved in the inhibition of the BCR/Abl-dependent proliferation signals in CML cells.

The link between energy shortage and environmental conditions where SC responsible for late relapse of disease are maintained underscores the interest for the characterization of the mechanisms of BCR/Abl suppression. We strongly believe that, under energy restriction, the balance within the CML cell population between clonal expansion and SC persistence is shifted towards the latter. As a consequence, proliferative stimuli such as those derived from BCR/Abl need to be suppressed within the SC environment [41]. The so-called “SC niche”, hematopoietic in particular, is a physiologically “hypoxic/ischemic” environment. In this context, the complex pattern of BCR/Abl protein suppression emerging from our results appears as an essential convergent aspect of CML cell adaptation to niche environment [42]. Cancer is indeed an evolutionary process at the cellular level, driven by stochastic genomic alterations which are selected by the interaction with microenvironment [43–45]. In this respect, it has been proposed that cancerogenesis is driven by a reverse evolution from multicellularity to unicellularity, where individual cancer cells increase their fitness to environment via the loss of multicellularity-related genetic constraints [46]. This is why evolution pushes cancer cells of different types and origins towards a convergent fate via different routes [42–47]. In conclusion, the concept emerging from our findings is that, in the course of CML cell adaptation to environmental metabolic constraints, BCR/Abl protein suppression is a mandatory phenomenon, driven via different routes as a common final target of the adaptation process.

BCR/Abl represents the “sole” oncogenic driver of CML [48] and one for which an extremely effective “biological” therapy -TKi- has been developed. However, minor CML cell subsets find their way to escape from TKi sensitivity, for a variety of reasons, including BCR/Abl protein suppression [49]. TKi are able in fact to target CML cell bulk, but not to eliminate SC of CML [50–53], well in keeping with BCR/Abl suppression in niche-adapted SC [4]. Thus, the cell line-specific BCR/Abl suppression mechanisms represent different routes converging to CML cell resistance to therapy. The post-translational mechanisms taken into consideration in our study have been shown involved in the resistance of SC of CML to TKi [54–57]. Furthermore, our findings hint to the possibility of using inhibitors of autophagy, apoptosis or proteasome to maintain BCR/Abl protein expression in LSC and thereby to target their adaptation to the SC niche. In this respect, it has been demonstrated, for instance, that autophagy acts as a survival signal in BCR/Abl-expressing cells treated with TKi and that its inhibition potentiates TKi-induced cell death, besides targeting the TKi-resistant SC of CML [58]. This may results in an improvement of current CML therapy aiming at the eradication of disease.

## MATERIALS AND METHODS

### Cells and culture conditions

Human K562 and KCL22 blast-crisis CML cell lines were purchased from German Collection of Cell Cultures (Braunschweig, Germany) and grown as previously described [12]. Full details are provided in the Supplementary Materials and Methods.

### Cell death/apoptosis

Determination of CML cell death/apoptosis was carried out by Annexin-V-FLUOS Staining kit (cat. 1858777, from Roche Diagnostics GmbH, Penzberg, Germany) according to the manufacturer's instructions. Briefly, cells (at a concentration of 1 × 10^6^ cells/mL) were washed with PBS and resuspended in 100 μL of Incubation buffer cointaining Annexin-V-Fluos labeling reagent and Propidium iodide (PI) solution and were incubated in the dark for 15 min at room temperature. Further 400 μL of Incubation buffer were added and the cells were analyzed immediately using a FACSCanto (Becton-Dickinson, Franklin Lakes, NJ, U.S.A.) flow cytometer. Data from at least 20000 events per sample were recorded and processed using BD FACSDiva™ software (Becton-Dickinson), and the % of Annexin V +/– and PI +/– cells were quantified using FlowJo software (FlowJo LLC, Ashland, OR, U.S.A.).

### Protein extraction and Western blotting

Proteins were extracted and separated essentially as previously described [12]. Full details of the protein extraction and Western blotting are provided in the Supplementary Materials and Methods. Primary antibodies used were: anti-cleaved-PARP (Asp214), rabbit polyclonal (cat. 9541), anti LC3 A/B, rabbit polyclonal (cat. 4108) (all from Cell Signaling Technology Danvers, MA, U.S.A.); anti-α-Tubulin (clone DM1A), mouse monoclonal (cat. T9026, from Sigma-Aldrich, St. Louis, MO, U.S.A.); anti-c-Abl (K-12), rabbit polyclonal (cat. sc-131), anti-ubiquitin (P4D1), mouse monoclonal (cat. sc-8017) (all from Santa Cruz Biotechnology, Santa Cruz, CA, U.S.A.); anti-puromycin mouse monoclonal (cat. MABE343, Merck Millipore). After washing with T-PBS, membranes were incubated for 1 h at RT in 1:1 Odyssey Blocking Buffer (LI-COR Biosciences, Lincoln, NE, U.S.A.)/PBS containing an IRDye^®^800CW- or IRDye^®^680-conjugated secondary antibody. Antibody-coated protein bands were visualized by the Odyssey Infrared Imaging System Densitometry and images analyzed by the Odyssey software to measure the mean fluorescence intensity value of the area selected for each band. A background measurement was also taken.

### Real time quantitative PCR

Total RNA was extracted using TRIzol reagent (Thermo Fisher Scientific, Waltham, MA, U.S.A.) according to the manufacturer's instructions. The concentration and purity of RNA were determined by absorbance at 260/280 nm, and 0.5 μg of RNA were used to generate cDNA using the ImProm-II^TM^ Reverse Transcription System (Promega, Madison, WI, U.S.A.) according to the manufacturer's protocol. Real Time quantitative PCR (qPCR) analysis of *BCR/abl* p210 transcript (b2a2 for KCL22 cells, b3a2 for K562 cells) was performed with the Applied Biosystems 7500 Fast Real-Time PCR System (Thermo Fisher Scientific): 2 min 95°C, 40 cycles at 95°C for 15 sec, 56°C for 20 sec, 60°C for 40 sec, using the GoTaq qPCR MasterMix (Promega). A melting curve analysis was performed to discriminate between specific and non-specific PCR products. Relative *BCR/abl* mRNA levels were normalized to different housekeeping genes, *β-actin*, *GAPDH*, *18S rRNA*, *GUSB*, *EIF2a* or *β-2* *microglobulin*, depending on the cell line and the metabolic condition. The sequences of oligonucleotide primers (all from Integrated DNA Technologies, Coralville, IA, U.S.A.) are shown in Supplementary Table S1.

### Analysis of mRNA stability

The half-life of *BCR-abl* mRNA was determined by treating K562 cells with 5μg/mL Actinomycin D (Sigma-Aldrich) to block transcription. During the following 8 h, cells were harvested every 2 hours and total RNA was extracted using TRIzol. The amounts of *BCR/abl* mRNA and *GAPDH* mRNA at each time point were determined by qPCR.

### Chromatin Immunoprecipitation (ChIP) assay

ChIP assay was performed essentially as previously described [59]. Full details of the ChIP assay are provided in the Supplementary Materials and Methods. ChIP-grade antibodies used (2μg) were: rabbit polyclonal anti-pan-acetylated-H4 (cat. 06–598, Merck-Millipore), rabbit IgG (cat. G5518, Sigma-Aldrich). The relative amount of immunoprecipitated *BCR* promoter DNA was determined by qPCR using the following primers (as described in [28]):

FWD 5′-CTGCGAGTTCTGCCAGAGAG-3′,
REV 5′-CACCCTCCCCCCGTCCCTGT-3′
The results were normalised by the fold-enrichment method and compared with the IgG-negative controls.

### SUnSET assay

The SUnSET assay is based on the use of puromycin, an aminonucleoside antibiotic produced by *S. alboniger*. Puromycin is incorporated into the nascent polypeptide chain and, when used in minimal amounts, its incorporation rate is proportional to mRNA translation *in vitro.* A monoclonal antibody to puromycin enables to directly monitor translation using a standard immunochemical method [16]. CML cells were incubated at 0.1% O_2_ in standard medium or at 21% O_2_ in the presence or the absence of glucose with the indicated concentrations of puromycin (cat. P8833, Sigma Aldrich) for 6 hours at different times of incubation. Cells were then collected and subjected to Western blotting as described above to assess rate of protein synthesis.

### Polysome profile analysis

Polysomes can be separated from free 80S ribosomes and ribosomal subunits by sucrose density gradient centrifugation, a method commonly used in translational control research [18]. A change in the association of mRNA with polysomes is indicative of changes in its translation state. Experimental procedures were performed as previously described [60]. Full details of the polysome profile analysis are provided in the Supplementary Materials and Methods.

Aliquots of cytoplasmic lysates were considered for sample comparison; “spike-in” *in vitro* transcribed *Renilla luciferase* transcript (1ng) was further added to each fraction, as suggested by [61], to normalize the relative yield after TRIzol RNA isolation. RNA samples were then analyzed by qPCR using *BCR/abl* and *Renilla luciferase* primers in an Applied Biosystems 7500 Fast Real-Time PCR System (Thermo Fisher Scientific): 15 min 37°C, 10 min 95°C, 42 cycles at 95°C for 10 sec, 56°C for 30 sec, 72°C for 30 sec, using the GoTaq 1-Step RT-qPCR System (Promega). The sequences of the used oligonucleotides are shown in Supplementary Table S1.

### Treatment with proteasome and/or caspase inhibitors

Cells were treated with the proteasome inhibitor MG132 (0.3 or 0.1 μM; cat. C2211, Sigma Aldrich) or the pan-caspase inhibitor z-VAD-fmk (50 μM; cat. 187389-52-2, MedChem Express, Stockholm, Sweden) and maintained under O_2_ or glucose shortage for 3 or 4 days, respectively. On day 2 of incubation, a half-dose of drug was added to culture. The combination of MG132 with Z-VAD-fmk was also tested (day 3). To assess the BCR/Abl protein level, Western blotting was then performed as described above.

### Treatment with autophagy inhibitors

Cells maintained under O_2_ or glucose shortage were treated with the late-stage autophagy inhibitor Bafilomycin A1 (2 nM; cat. B1793, Sigma Aldrich) at different times of incubation. As positive control of autophagic flux inhibition, a very high dose of Chloroquine was used (100 μM for 24 hours; cat. C6628, Sigma Aldrich). To assess the LC3 I/II and BCR/Abl protein level, Western blotting was performed as described above.

### Immunofluorescence and confocal microscopy

Cells (1 × 10^5^) were spun on microscope slides (Menzel-Gläser, by Thermo Fisher Scientific) at 800 rpm for 6 min in a cytocentrifuge (Aerospray Pro slide stainer and cytocentrifuge 7152, Delcon, Milan, Italy). Slides were fixed for 15 min with ice-cold 100% methanol, rinsed 3 times with PBS, blocked with 5% horse serum and 0.3% Triton^®^ X-100 (VWR) in PBS, and incubated with LC3 A/B antibody overnight at 4°C. Slides were then washed 3 times with PBS and incubated with a Cy2-conjugated secondary anti-rabbit antibody (cat. AP132J, Merck-Millipore) for 1 h at room temperature. Nuclei were counterstained by the Hoechst 33342 stain (cat. B2261, Sigma Aldrich). Cells were then dried and examined with a Nikon Eclipse TE2000-U confocal microscope (Nikon, Tokyo, Japan). A single image was obtained by superimposition of 10 optical sections at 63x magnification for each sample using the ImageJ software (developed by Wayne Rasband, National Institutes of Health, Bethesda, MD, U.S.A. and available at http://rsbweb.nih.gov/ij/index.html).

### Statistical analysis

All data are presented as the mean ± SD (unless indicated otherwise) of independent experiments and were compared by using the Student's *t* test. *P* values (p) of ≤ 0.05 were considered statistically significant.

## SUPPLEMENTARY MATERIALS FIGURES AND TABLES



## References

[1] Neviani P, Harb JG, Oaks JJ, Santhanam R, Walker CJ, Ellis JJ, Ferenchak G, Dorrance AM, Paisie CA, Eiring AM, Ma Y, Mao HC, Zhang B (2013). PP2A-activating drugs selectively eradicate TKI-resistant chronic myeloid leukemic stem cells. J Clin Invest.

[2] Taverna S, Giallombardo M, Pucci M, Flugy A, Manno M, Raccosta S, Rolfo C, De Leo G, Alessandro R (2015). Curcumin inhibits *in vitro* and *in vivo* chronic myelogenous leukemia cells growth: a possible role for exosomal disposal of miR-21. Oncotarget.

[3] Ng KP, Manjeri A, Lee KL, Huang W, Tan SY, Chuah CT, Poellinger L, Ong ST (2014). Physiologic hypoxia promotes maintenance of CML stem cells despite effective BCR-ABL1 inhibition. Blood.

[4] Rovida E, Peppicelli S, Bono S, Bianchini F, Tusa I, Cheloni G, Marzi I, Cipolleschi MG, Calorini L, Sbarba PD (2014). The metabolically-modulated stem cell niche: a dynamic scenario regulating cancer cell phenotype and resistance to therapy. Cell Cycle.

[5] Benito J, Zeng Z, Konopleva M, Wilson WR (2013). Targeting hypoxia in the leukemia microenvironment. Int J Hematol Oncol.

[6] Cipolleschi MG, Dello Sbarba P, Olivotto M (1993). The role of hypoxia in the maintenance of hematopoietic stem cells. Blood.

[7] Ivanovic Z (2009). Hypoxia or *in situ* normoxia: The stem cell paradigm. J Cell Physiol.

[8] Ivanovic Z, Belloc F, Faucher JL, Cipolleschi MG, Praloran V, Dello Sbarba P (2002). Hypoxia maintains and interleukin-3 reduces the pre-colony-forming cell potential of dividing CD34(+) murine bone marrow cells. Exp Hematol.

[9] Giuntoli S, Rovida E, Gozzini A, Barbetti V, Cipolleschi MG, Olivotto M, Dello Sbarba P (2007). Severe hypoxia defines heterogeneity and selects highly immature progenitors within clonal erythroleukemia cells. Stem Cells.

[10] Eliasson P, Jonsson JI (2010). The hematopoietic stem cell niche: low in oxygen but a nice place to be. J Cell Physiol.

[11] Giuntoli S, Rovida E, Barbetti V, Cipolleschi MG, Olivotto M, Dello Sbarba P (2006). Hypoxia suppresses BCR/Abl and selects imatinib-insensitive progenitors within clonal CML populations. Leukemia.

[12] Giuntoli S, Tanturli M, Di Gesualdo F, Barbetti V, Rovida E, Dello Sbarba P (2011). Glucose availability in hypoxia regulates the selection of chronic myeloid leukemia progenitor subsets with different resistance to imatinib-mesylate. Haematologica.

[13] Marega M, Piazza RG, Pirola A, Redaelli S, Mogavero A, Iacobucci I, Meneghetti I, Parma M, Pogliani EM, Gambacorti-Passerini C (2010). BCR and BCR-ABL regulation during myeloid differentiation in healthy donors and in chronic phase/blast crisis CML patients. Leukemia.

[14] Shahbazian MD, Grunstein M (2007). Functions of site-specific histone acetylation and deacetylation. Annu Rev Biochem.

[15] Eberharter A, Becker PB (2002). Histone acetylation: a switch between repressive and permissive chromatin. Second in review series on chromatin dynamics. EMBO reports.

[16] Schmidt EK, Clavarino G, Ceppi M, Pierre P (2009). SUnSET, a nonradioactive method to monitor protein synthesis. Nat Methods.

[17] Bowling H, Zhang G, Bhattacharya A, Perez-Cuesta LM, Deinhardt K, Hoeffer CA, Neubert TA, Gan WB, Klann E, Chao MV (2014). Antipsychotics activate mTORC1-dependent translation to enhance neuronal morphological complexity. Sci Signal.

[18] Preiss T, Baron-Benhamou J, Ansorge W, Hentze MW (2003). Homodirectional changes in transcriptome composition and mRNA translation induced by rapamycin and heat shock. Nat Struct Biol.

[19] Giuliano S, Cormerais Y, Dufies M, Grepin R, Colosetti P, Belaid A, Parola J, Martin A, Lacas-Gervais S, Mazure NM, Benhida R, Auberger P, Mograbi B (2015). Resistance to sunitinib in renal clear cell carcinoma results from sequestration in lysosomes and inhibition of the autophagic flux. Autophagy.

[20] Mizushima N, Yoshimori T (2014). How to Interpret LC3 Immunoblotting. Autophagy.

[21] Helgason GV, Karvela M, Holyoake TL (2011). Kill one bird with two stones: potential efficacy of BCR-ABL and autophagy inhibition in CML. Blood.

[22] Fontana S, Alessandro R, Barranca M, Giordano M, Corrado C, Zanella-Cleon I, Becchi M, Kohn EC, De Leo G (2007). Comparative proteome profiling and functional analysis of chronic myelogenous leukemia cell lines. J Proteom Res.

[23] Shah NP, Witte ON, Denny CT (1991). Characterization of the BCR promoter in Philadelphia chromosome-positive and -negative cell lines. Mol Cell Biol.

[24] Jamieson CH, Ailles LE, Dylla SJ, Muijtjens M, Jones C, Zehnder JL, Gotlib J, Li K, Manz MG, Keating A, Sawyers CL, Weissman IL (2004). Granulocyte-macrophage progenitors as candidate leukemic stem cells in blast-crisis CML. N Engl J Med.

[25] Yang X, Pang J, Shen N, Yan F, Wu LC, Al-Kali A, Litzow MR, Peng Y, Lee RJ, Liu S (2016). Liposomal bortezomib is active against chronic myeloid leukemia by disrupting the Sp1-BCR/ABL axis. Oncotarget.

[26] Sharma N, Magistroni V, Piazza R, Citterio S, Mezzatesta C, Khandelwal P, Pirola A, Gambacorti-Passerini C (2015). BCR/ABL1 and BCR are under the transcriptional control of the MYC oncogene. Mol Cancer.

[27] Brusa G, Zuffa E, Mancini M, Benvenuti M, Calonghi N, Barbieri E, Santucci MA (2006). P210 Bcr-abl tyrosine kinase interaction with histone deacetylase 1 modifies histone H4 acetylation and chromatin structure of chronic myeloid leukaemia haematopoietic progenitors. Br J Haematol.

[28] Qin R, Li K, Qi X, Zhou X, Wang L, Zhang P, Zou L (2014). beta-Arrestin1 promotes the progression of chronic myeloid leukaemia by regulating BCR/ABL H4 acetylation. Br J Cancer.

[29] Dang CV, Le A, Gao P (2009). MYC-induced cancer cell energy metabolism and therapeutic opportunities. Clin Cancer Res.

[30] Cummins EP, Taylor CT (2005). Hypoxia-responsive transcription factors. Pflugers Arch.

[31] Buttgereit F, Brand MD (1995). A hierarchy of ATP-consuming processes in mammalian cells. Biochem J.

[32] Spriggs KA, Bushell M, Willis AE (2010). Translational regulation of gene expression during conditions of cell stress. Mol Cell.

[33] Walters B, Thompson SR (2016). Cap-Independent Translational Control of Carcinogenesis. Front Oncol.

[34] Tsukahara F, Maru Y (2010). Bag1 directly routes immature BCR-ABL for proteasomal degradation. Blood.

[35] Chen YJ, Chang LS (2012). Gallic acid downregulates matrix metalloproteinase-2 (MMP-2) and MMP-9 in human leukemia cells with expressed Bcr/Abl. Mol Nutr Food Res.

[36] Di Bacco AM, Cotter TG (2002). p53 expression in K562 cells is associated with caspase-mediated cleavage of c-ABL and BCR-ABL protein kinases. Br J Haematol.

[37] Wojcik C (2002). Regulation of apoptosis by the ubiquitin and proteasome pathway. J Cell Mol Med.

[38] Almond JB, Snowden RT, Hunter A, Dinsdale D, Cain K, Cohen GM (2001). Proteasome inhibitor-induced apoptosis of B-chronic lymphocytic leukaemia cells involves cytochrome c release and caspase activation, accompanied by formation of an approximately 700 kDa Apaf-1 containing apoptosome complex. Leukemia.

[39] McLaughlin B (2004). The kinder side of killer proteases: caspase activation contributes to neuroprotection and CNS remodeling. Apoptosis.

[40] Altman BJ, Jacobs SR, Mason EF, Michalek RD, MacIntyre AN, Coloff JL, Ilkayeva O, Jia W, He YW, Rathmell JC (2011). Autophagy is essential to suppress cell stress and to allow BCR-Abl-mediated leukemogenesis. Oncogene.

[41] Del Poggetto E, Tanturli M, Ben-Califa N, Gozzini A, Tusa I, Cheloni G, Marzi I, Cipolleschi MG, Kashman Y, Neumann D, Rovida E, Dello Sbarba P (2015). Salarin C inhibits the maintenance of chronic myeloid leukemia progenitor cells. Cell Cycle.

[42] Olivotto M, Dello Sbarba P (2008). Environmental restrictions within tumor ecosystems select for a convergent, hypoxia-resistant phenotype of cancer stem cells. Cell Cycle.

[43] Nowell PC (1976). The clonal evolution of tumor cell populations. Science.

[44] Greaves M (2015). Evolutionary determinants of cancer. Cancer Discov.

[45] Danielsen HE, Pradhan M, Novelli M (2016). Revisiting tumour aneuploidy - the place of ploidy assessment in the molecular era. Nat Rev Clin Oncol.

[46] Chen H, Lin F, Xing K, He X (2015). The reverse evolution from multicellularity to unicellularity during carcinogenesis. Nat Commun.

[47] Chen H, He X (2016). The Convergent Cancer Evolution toward a Single Cellular Destination. Mol Biol Evol.

[48] Appelmann I, Rillahan CD, de Stanchina E, Carbonetti G, Chen C, Lowe SW, Sherr CJ (2015). Janus kinase inhibition by ruxolitinib extends dasatinib- and dexamethasone-induced remissions in a mouse model of Ph+ ALL. Blood.

[49] Cipolleschi MG, Rovida E, Dello Sbarba P (2013). The Culture-Repopulating Ability assays and incubation in low oxygen: a simple way to test drugs on leukaemia stem or progenitor cells. Curr Pharm Des.

[50] Corbin AS, Agarwal A, Loriaux M, Cortes J, Deininger MW, Druker BJ (2011). Human chronic myeloid leukemia stem cells are insensitive to imatinib despite inhibition of BCR-ABL activity. J Clin Invest.

[51] Holyoake TL, Helgason GV (2014). Do we need more drugs for chronic myeloid leukemia?. Immunol Rev.

[52] Gorre ME, Mohammed M, Ellwood K, Hsu N, Paquette R, Rao PN, Sawyers CL (2001). Clinical resistance to STI-571 cancer therapy caused by BCR-ABL gene mutation or amplification. Science.

[53] Copland M, Hamilton A, Elrick LJ, Baird JW, Allan EK, Jordanides N, Barow M, Mountford JC, Holyoake TL (2006). Dasatinib (BMS-354825) targets an earlier progenitor population than imatinib in primary CML but does not eliminate the quiescent fraction. Blood.

[54] Heaney NB, Pellicano F, Zhang B, Crawford L, Chu S, Kazmi SM, Allan EK, Jorgensen HG, Irvine AE, Bhatia R, Holyoake TL (2010). Bortezomib induces apoptosis in primitive chronic myeloid leukemia cells including LTC-IC and NOD/SCID repopulating cells. Blood.

[55] Bucur O, Stancu AL, Goganau I, Petrescu SM, Pennarun B, Bertomeu T, Dewar R, Khosravi-Far R (2013). Combination of bortezomib and mitotic inhibitors down-modulate Bcr-Abl and efficiently eliminates tyrosine-kinase inhibitor sensitive and resistant Bcr-Abl-positive leukemic cells. PLoS One.

[56] Crawford LJ, Chan ET, Aujay M, Holyoake TL, Melo JV, Jorgensen HG, Suresh S, Walker B, Irvine AE (2014). Synergistic effects of proteasome inhibitor carfilzomib in combination with tyrosine kinase inhibitors in imatinib-sensitive and -resistant chronic myeloid leukemia models. Oncogenesis.

[57] Fulda S (2013). Regulation of apoptosis pathways in cancer stem cells. Cancer Lett.

[58] Bellodi C, Lidonnici MR, Hamilton A, Helgason GV, Soliera AR, Ronchetti M, Galavotti S, Young KW, Selmi T, Yacobi R, Van Etten RA, Donato N, Hunter A (2009). Targeting autophagy potentiates tyrosine kinase inhibitor-induced cell death in Philadelphia chromosome-positive cells, including primary CML stem cells. J Clin Invest.

[59] Barbetti V, Tusa I, Cipolleschi MG, Rovida E, Dello Sbarba P (2013). AML1/ETO sensitizes via TRAIL acute myeloid leukemia cells to the pro-apoptotic effects of hypoxia. Cell death & disease. Cell Death Dis.

[60] D'Agostino VG, Lal P, Mantelli B, Tiedje C, Zucal C, Thongon N, Gaestel M, Latorre E, Marinelli L, Seneci P, Amadio M, Provenzani A (2015). Dihydrotanshinone-I interferes with the RNA-binding activity of HuR affecting its post-transcriptional function. Sci Rep.

[61] Clancy JL, Nousch M, Humphreys DT, Westman BJ, Beilharz TH, Preiss T (2007). Methods to Analyze MicroRNA-Mediated Control of mRNA Translation. Methods Enzymol.

