# Supplementary material for: Different BCR/Abl protein suppression patterns as a converging trait of chronic myeloid leukemia cell adaptation to energy restriction
Source: Oncotarget. 2016 Nov 12;7(51):84810–25. doi: 10.18632/oncotarget.13319 (PMC5356700; doi:10.18632/oncotarget.13319)
Supplement: Supplementary file 1 [file oncotarget-07-84810-s001.pdf]

Different BCR/Abl protein suppression patterns as a converging trait of chronic myeloid leukemia cell adaptation to energy restriction

Supplementary Materials

SUPPLEMENTARY MATERIALS AND METHODS

Cell culture

Proteins were extracted and separated as previously described [1]. Cells were cultured in RPMI 1640 medium containing 2 g/D-glucose and supplemented with 10% heat-inactivated foetal bovine serum, 2 mM L-glutamine, 50 units/mL penicillin and 50 µg/ml streptomycin (all from EuroClone, Paington, U.K.). In some experiments, we used RPMI 1640 medium without D-glucose (Gibco by Thermo Fisher Scientific, Waltham, MA, U.S.A.), supplemented as above. Experiments were performed with cells harvested from exponentially-growing maintenance cultures and subcultured ($5x10^5$/mL) in fresh medium 24 h before plating ($3x10^5$/mL). Incubation was carried out at 37°C in water-saturated atmosphere containing 5% CO_2 and 21% O_2 ("standard atmosphere"), in a conventional cell culture incubator, or 0.1% O_2 ("low oxygen") in a gas-tight incubator/manipulator (Don Whitley Scientific DG250 anaerobic workstation), flushed with a preformed gas mixture (0.1% O_2, 5% CO_2, 94.9% N_2). Viable cells were counted in a hemocytometer by trypan blue exclusion.

Protein extraction and Western blotting

Proteins were extracted and separated as previously described [1]. Briefly, cells were washed once with ice-cold phosphate buffered saline (PBS) containing 100 µM Na_3VO_4 and solubilized by incubating for 10 minutes at 95°C in Laemmli buffer (62.5 mM Tris/HCl, pH 6.8, 10% glycerol, 0.005% bromophenol blue, and 2% SDS). Lysates were clarified by centrifugation (13000 rpm, 5 minutes, RT) and protein concentration in supernatants was determined by the BCA method (Pierce™ BCA Protein Assay Kit; Thermo Fisher Scientific). Extracted proteins (50 µg/sample) were boiled for 10 minutes in the presence of 100 mM 2-mercaptoethanol, separated by SDS-PAGE in 9 or 15% polyacrylamide minigels and then transferred onto PVDF membranes (Merck-Millipore, Billerica, MA, U.S.A.) by electroblotting. Membranes were blocked in a 1:1 dilution of Odyssey (LI-COR® Biosciences, Lincoln, NE, U.S.A.) blocking buffer (OBB) with PBS for 1 hour at RT and then incubated (overnight, 4°C) with primary antibody in a 1:1 dilution of OBB with PBS-0,1% Tween (T-PBS).

(ChIP) assay

ChIP assay procedure was performed as previously described [2]. One million K562 cells were treated with 1% formaldehyde for 10 min at 37°C and centrifuged at 1100 rpm for 5 min at 4°C. Cells were then washed twice, using ice-cold PBS containing protease inhibitors: 100 µM Na_3VO_4, 1 mM phenyl-methyl-sulphonyl-fluoride (PMSF), 1 µg/ml aprotinin and 1 µg/ml pepstatin-A. Cell pellet was lysed in 200 µl of lysis buffer (50 mM Tris–HCl pH 8.1, 10 mM EDTA, 1% SDS, 100 µM Na_3VO_4,1 mM PMSF, 1 µg/ml aprotinin and 1 µg/ml pepstatin-A) for 10 min on ice and then sonicated (180 pulses, 15 s on and 28 s off each, at maximum power in a Sonoplus Bandelin GM3200 apparatus) to generate DNA fragments of 100-500 bp. After centrifugation (13000 rpm for 10 min, 4°C), the supernatant was 10-fold diluted with ChIP dilution buffer (16.7 mM Tris-HCl pH 8.1, 167 mM NaCl, 1.2 mM EDTA, 0.01% SDS, 1.1% Triton X-100), 4% of this sample was harvested and used as an indicator of chromatin content in each sample (input). The samples were pre-cleared by incubating with 200 µl of PBS/0.02% Tween and 14 µl of Dynabeads® Protein G (cat. 10003D, Thermo Fisher Scientific) for 1 h at 4°C under constant rotation. The bead-antibody complexes were recovered with a Dyna Mag™-2 Magnet (cat. 12321D, Thermo Fisher Scientific), resuspended in 100 µl PBS/BSA 100 mg/ml and added to each sample to be incubated for 16-18 h at 4°C under constant rotation. In parallel, each sample was subjected to the same procedure without antibody (negative control). The supernatant was removed and, after extensive washing, antibody-protein-DNA complexes were eluted from beads with 500 µl elution buffer (0.1 M $NaHCO_3$, 1% SDS). Following addition of 0.2 M NaCl, all samples, including input, were incubated for 4 h at 65°C to revert cross-linking. After treatment with 10 mM RNAase and digestion with 40 mM proteinase-K, DNA was extracted using QIAcquick PCR purification kit, according to the manufacturer's recommendations (cat. 28106, Qiagen, German-Town, MD, U.S.A.) and DNA eluted in 40 µl of 10 mM Tris-HCl/1mM EDTA pH 8.0 (TE) buffer.

Polysome profile analysis

Experimental procedures were performed as previously described [3]. K562 and KCL22 cells (2×10^7) were incubated at 0.1% O_2 in standard medium or at 21% O_2 in the absence of glucose for different times. Cycloheximide was added to a final concentration of 0.1 mg/mL for 15 min at 37°C before cells harvesting. After washing with PBS and centrifugation, cytoplasmic cell lysates were obtained by resuspending pellets, on ice, in a buffer containing 20 mM Tris-HCl pH 7.5, 100 mM NaCl, 5 mM $MgCl_2$, 1% Triton X-100, 1% sodium deoxycholate, 1 mM DTT, 10 µg/mL cycloheximide, 100 U/mL RNAse inhibitor. After 15 min of incubation, nuclei were pelleted by centrifugation at 10000*g* at 4°C. Soluble cytoplasmic extracts, containing approximately 300 µg RNA [4], were subjected to 15-50% sucrose gradient centrifugation at 40000 rpm in an OPTIMA XPN ultracentrifuge (Beckman Coulter, Brea, CA, U.S.A.). Samples were then fractionated using a TELEDYNE ISCO system (TELEDYNE ISCO, Lincoln, NE, U.S.A) and their absorbance at 254 nm recorded.

SUPPLEMENTARY REFERENCES

1. Giuntoli S, Tanturli M, Di Gesualdo F, Barbetti V, Rovida E, Dello Sbarba P. Glucose availability in hypoxia regulates the selection of chronic myeloid leukemia progenitor subsets with different resistance to imatinib-mesylate. Haematologica. 2011; 96:204–212.
2. Barbetti V, Tusa I, Cipolleschi MG, Rovida E, Dello Sbarba P. AML1/ETO sensitizes via TRAIL acute myeloid leukemia cells to the pro-apoptotic effects of hypoxia. Cell death dis. 2013; 4:e536.
3. D'Agostino VG, Lal P, Mantelli B, Tiedje C, Zucal C, Thongon N, Gaestel M, Latorre E, Marinelli L, Seneci P, Amadio M, Provenzani A. Dihydrotanshinone-I interferes with the RNA-binding activity of HuR affecting its post-transcriptional function. Sci Rep. 2015; 5:16478.
4. Zucal C, D'Agostino VG, Casini A, Mantelli B, Thongon N, Soncini D, Caffa I, Cea M, Ballestrero A, Quattrone A, Indraccolo S, Nencioni A, Provenzani A. EIF2A-dependent translational arrest protects leukemia cells from the energetic stress induced by NAMPT inhibition. BMC Cancer. 2015; 15:855.

Supplementary Table S1: Primer sequences for q-PCR

Target gene	Primer	Sequence
BCR/abl	Forward	5′-GGAGCAGCAGAAGAAGTGTTT-3′
	Reverse	5′-TGGGTCCAGCGAGAAGGTTTT-3′
β-actin	Forward	5′-GAAACTACCTTCAACTCCATCATG-3′
	Reverse	5′-AGGAGGAGCAATGATCTTGATC-3′
GAPDH	Forward	5′-AACAGCCTCAAGATCATCAGCAA-3′
	Reverse	5′-TCTGGGTGGCAGTGAT-3′
18S	Forward	5′-CGGCTACCACATCCAAGGAA-3′
	Reverse	5′-GCTGGAATTACCGCGGCT-3′
GUSB	Forward	5′-GAAAATATGTGGTTGGAGAGCTCATT-3′
	Reverse	5′-CCGAGTGAAGATCCCCTTTTTA-3′
EIF2a	Forward	5′-GGATGGGACCTTGTTTGCCT-3′
	Reverse	5′-CCACGTTGCCAGGACAGTAT-3′
β-2 microglobulin	Forward	5′-AGTATGCCTGCCGTGTGAAC-3′
	Reverse	5′-GCGGCATCTTCACAAACCTCCA-3′
Renilla luc	Forward	5′-GTCGAGACCATGCTCCCAGCA-3′
	Reverse	5′-TTGCGGACAATCTGGACGACGT-3′

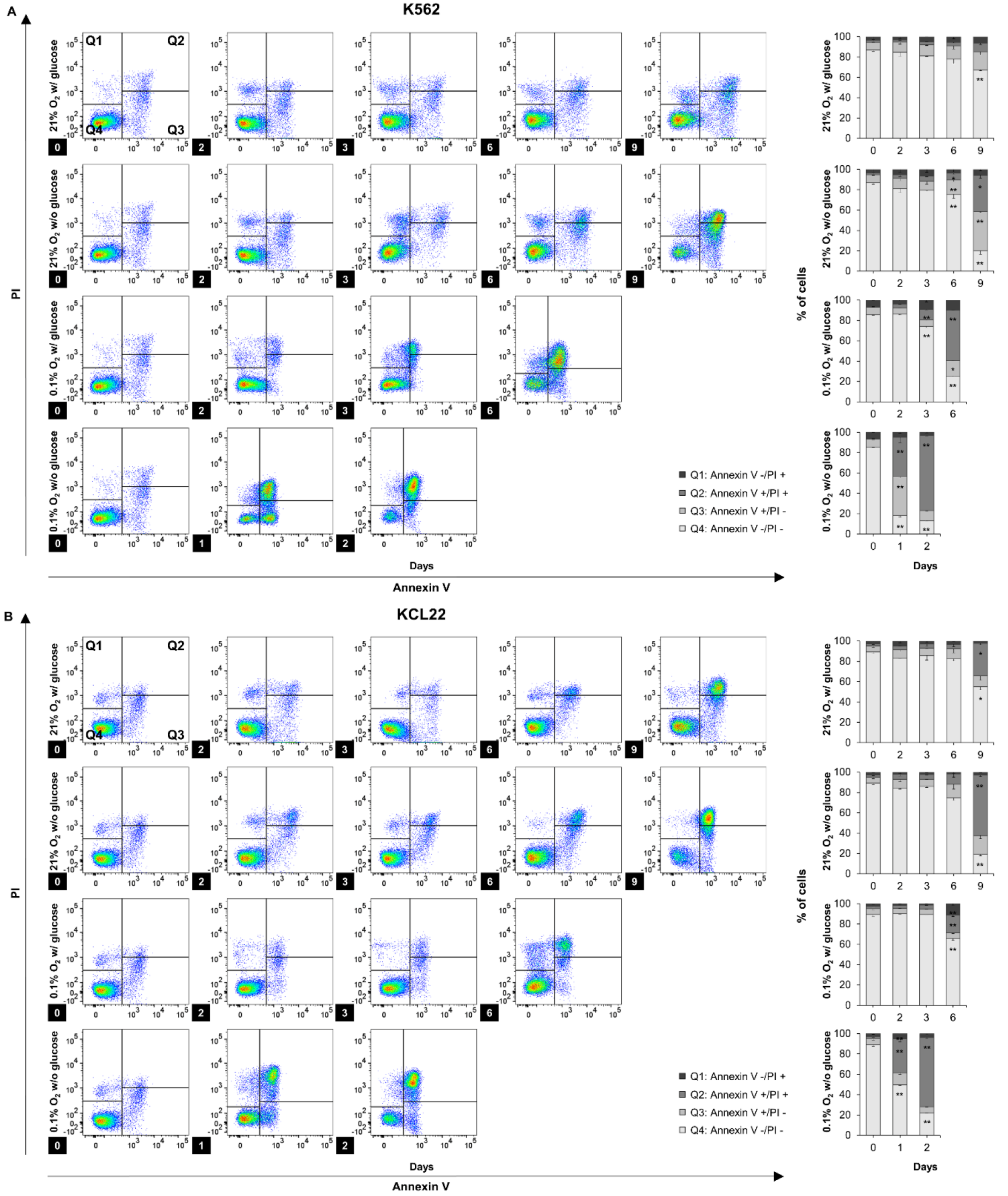

Supplementary Figure S1: Cell death/apoptosis. K562 (**A**) or KCL22 (**B**) cells were plated at 3x10^{5} cells/mL and incubated at 21% O_2 w/ glucose or 21% O_2 w/o glucose, or at 0.1% O_2 w/ glucose or 0.1% O_2 w/o glucose for the indicated times. Representative flow cytometry dot plot of Annexin V/PI staining (left panels). Data were quantified using FlowJo software; gates were opportunely set based on single (Annexin V or PI) staining. Q1: necrotic cells; Q2: late apoptotic cells; Q3: early apoptotic cells; Q4: viable cells. The histograms (right panels) represent the mean - SD of 3 independent experiments; **$p \leq 0.01$, *$p \leq 0.05$ compared with time 0 (two-tailed Student's *t* test).

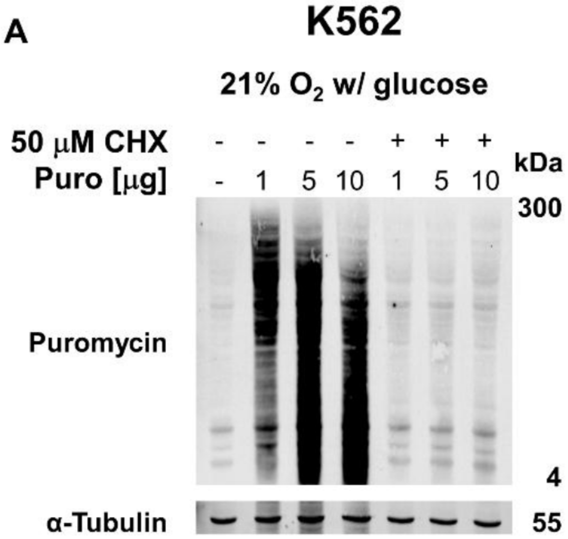

Supplementary Figure S2: Determination of the optimal concentration of puromycin. K562 (**A**) or KCL22 (**B**) cells were incubated at 21% O_2 in standard medium with 1, 5, or 10 μg/mL of puromycin for 6 hours, in the presence or the absence of 50 μM cycloheximide (CHX), as indicated. Cell extracts were separated by denaturing electrophoresis and analyzed by Western blotting using a monoclonal antibody against puromycin (12D10); α-Tubulin was used as loading control. On this basis, 1 μg/mL puromycin was chosen for the experiments of Figure 5A and B, as it yielded a maximum of high molecular weight protein bands (low molecular weight proteins, indeed, indicate incomplete translation due to the puromycin inhibition of ribosome). The results are representative of multiple independent experiments.

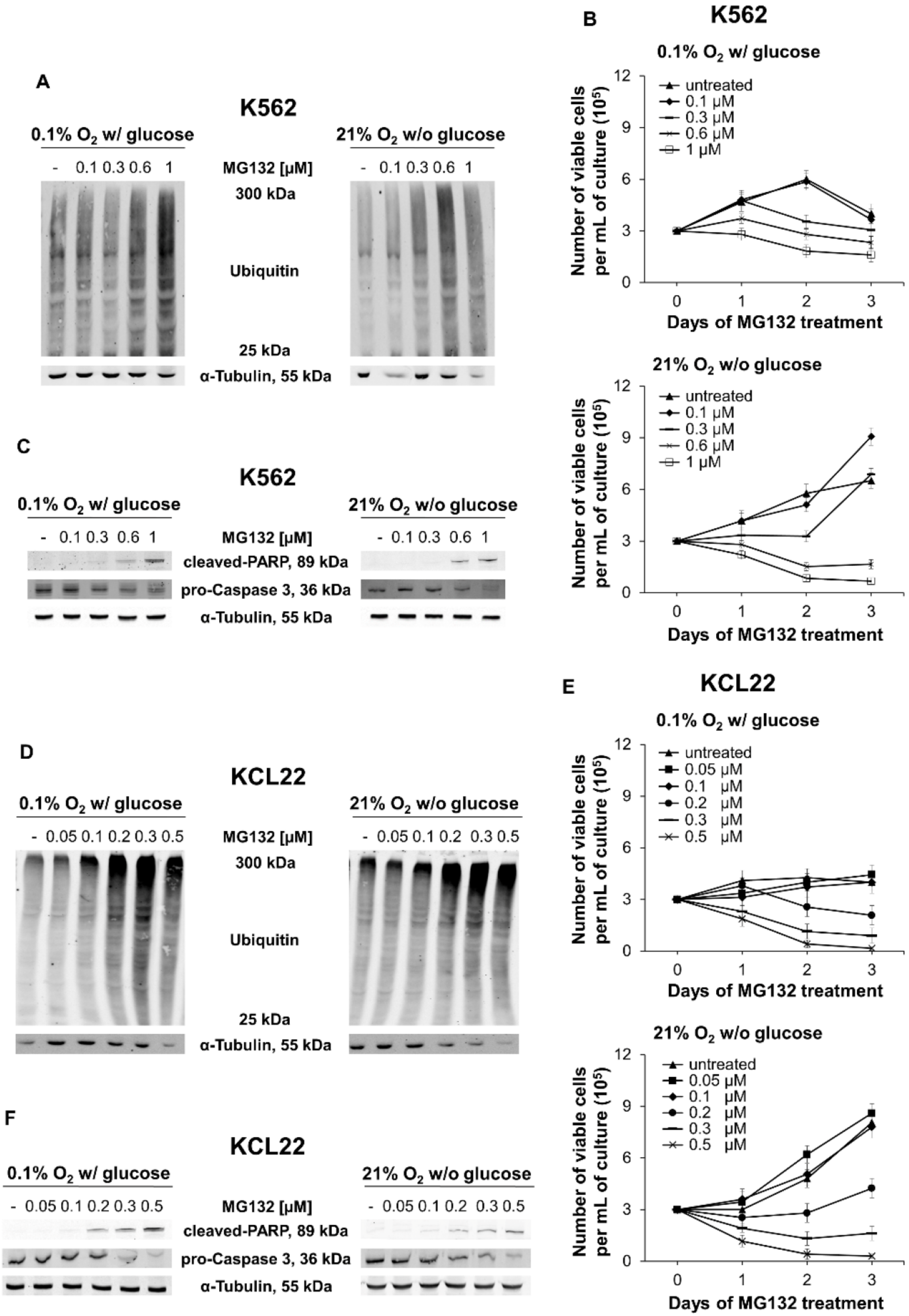

Supplementary Figure S3: Determination of the optimal MG132 concentrations. K562 (**A**–**C**) or KCL22 (**D**–**F**) cells were incubated at 0.1% O_2 in standard medium or at 21% O_2 in the absence of glucose in the presence of the indicated concentrations of MG132. Cell lysates were subjected to Western blotting using anti-Ubiquitin (A and D) or anti-cleaved-PARP and anti-pro-Caspase 3 (C and F) antibodies; α-Tubulin was used as loading control. Growth curves show data (mean ± SD) obtained from 3 independent experiments (B and E)**.** Results shown in A, C, D, and F are representative of multiple independent experiments.

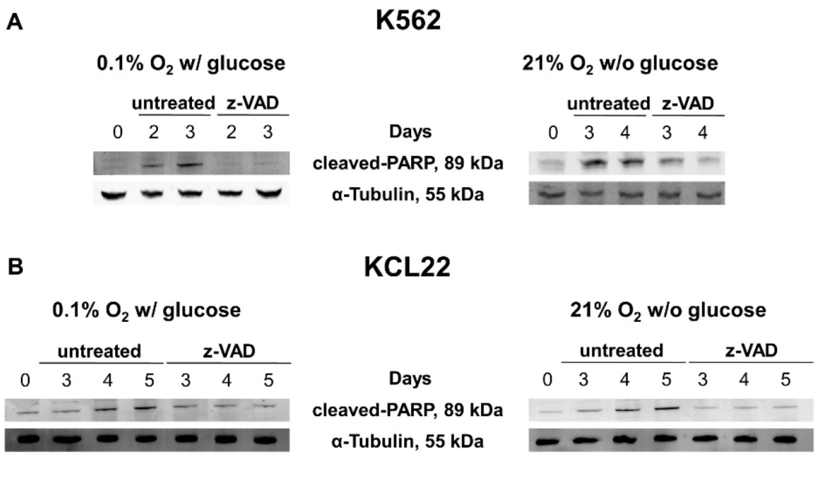

Supplementary Figure S4: Effects of z-VAD-fmk on apoptosis induction under oxygen or glucose shortage. K562 (**A**) or KCL22 (**B**) cells were incubated at 0.1% O_2 in standard medium (left panels) or at 21% O_2 in the absence of glucose (right panels), and treated with the pan-caspase inhibitor z-VAD-fmk (50 µM) for the indicated times. Cleaved-PARP was detected by Western blotting, using α-Tubulin as loading control. The results are representative of multiple independent experiments.

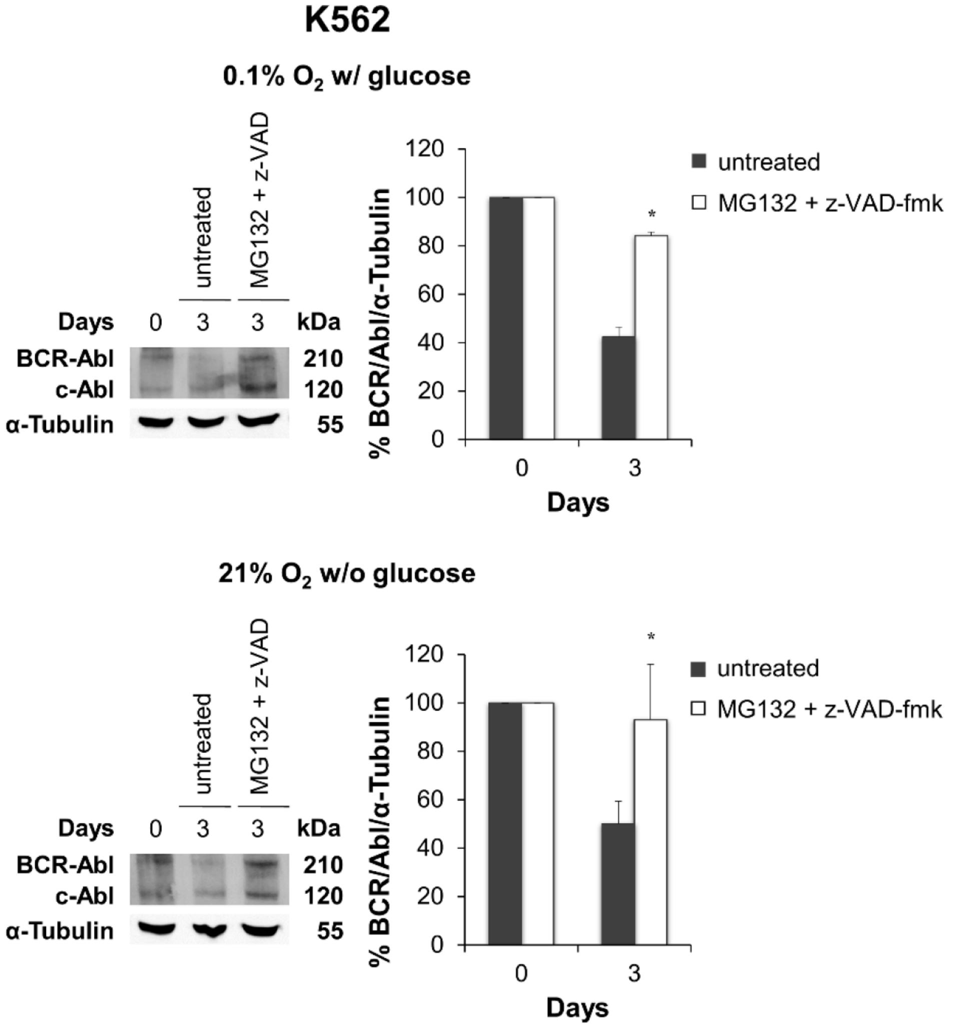

Supplementary Figure S5: Effects of the z-VAD-fmk/MG132 combination on BCR/Abl protein expression under oxygen or glucose shortage. K562 cells were incubated at 0.1% O_2 in standard medium (top panel) or at 21% O_2 in the absence of glucose (bottom panel), treated with both MG132 and z-VAD-fmk for 3 days and lysed. BCR/Abl protein expression was determined by Western blotting, using α-Tubulin as loading control. Band intensity was quantified using the Odyssey software. Data were normalized with respect to the corresponding α-Tubulin band intensity and expressed as percentage of time 0 (day 0) value. Histograms represent the mean + SD of 3 independent experiments; $*p \leq 0.05$ compared with untreated (two-tailed Student's *t* test).

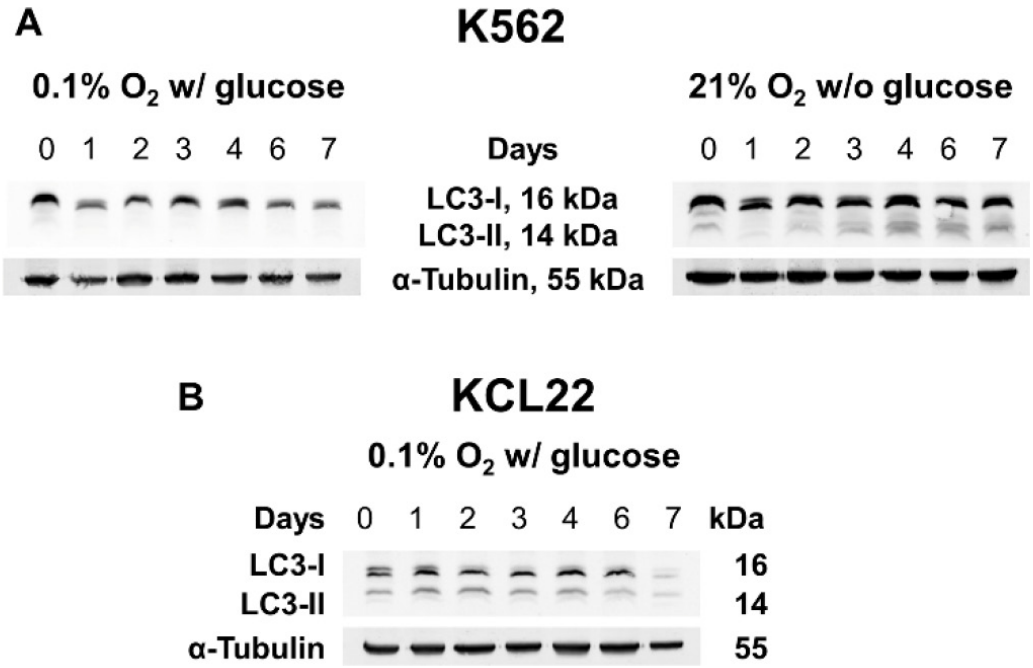

Supplementary Figure S6: Analysis of LC3 protein levels under oxygen or glucose shortage. K562 (**A**) or KCL22 (**B**) cells were incubated at 0.1% O_2 in standard medium (A, left panel, and B) or at 21% O_2 in the absence of glucose (A, right panel) for the indicated times. LC3-I and LC3-II levels were determined by Western blotting; α-Tubulin was used as loading control. The results are representative of multiple independent experiments.
